# Idiopathic inflammatory myopathies overlapping with systemic diseases 

**DOI:** 10.5414/NP301077

**Published:** 2017-11-20

**Authors:** Sébastien Lepreux, Johannes A. Hainfellner, Anne Vital

**Affiliations:** 1 Pathology Department, Bordeaux University Hospital, Bordeaux, France,; 2 Institute of Neurology and Comprehensive Cancer Center CNS Unit, Medical University of Vienna, Austria, and; 3 Univ. Bordeaux, Institut des Maladies Neurodégénératives, UMR 5293, Bordeaux, France

**Keywords:** idiopathic inflammatory myopathy, connective tissue disease, muscle biopsy

## Abstract

A muscle biopsy is currently requested to assess the diagnosis of an idiopathic inflammatory myopathy overlapping with a systemic disease. During the past few years, the classification of inflammatory myopathy subtypes has been revisited progressively on the basis of correlations between clinical phenotypes, autoantibodies and histological data. Several syndromic entities are now more clearly defined, and the aim of the present review is to clarify the contribution of muscle biopsy in a setting of idiopathic inflammatory myopathies overlapping with systemic diseases.

## Introduction 

The increasing evidence of a high frequency of idiopathic inflammatory myopathies in a setting of overlapping connective tissue diseases suggested the use of novel classification criteria including “overlap myositis” as a distinct entity and the use of overlap autoantibodies as a tool for diagnosis [1]. The connective diseases that are classically overlapping with myositis are systemic sclerosis, systemic lupus erythematosus, Sjögren syndrome, and rheumatoid arthritis [2], but other clinical features, especially interstitial lung disease (ILD), are important criteria for delimitation of respective disorders. Idiopathic inflammatory myopathies are associated with a large panel of autoantibodies that are directed toward defined nuclear and cytoplasmic antigens [3]. Some of these autoantibodies are frequently detected in patients with connective tissue diseases overlapping myositis, especially systemic sclerosis, and are referred to as myositis-associated autoantibodies (MAAs). Other autoantibodies are considered specific to inflammatory myopathies and called myositis specific antibodies (MSAs) including autoantibodies recognizing a subset of aminoacyl-tRNA synthetase (ARS). An increased risk of interstitial lung disease has been reported in patients with myositis who are positive for anti-ARS, an association often referred to as the antisynthetase syndrome [4, 5, 6]. In fact, the populations delineated by MAAs and MSAs may overlap. In most patients, only one MSA is identified, whereas MAAs can be found in multiple combinations and accompanying MSA [7, 8]. Moreover, there appears to be considerable racial and geographic variation in the frequency of MSAs and the associated clinical phenotypes [9]. 

Although muscle biopsy is considered the gold standard for the diagnosis of myositis, the reported histological studies in a setting of overlapping connective tissue diseases did not allow definite conclusion when not correlated with the combined use of autoantibodies. With correlations between clinical phenotypes, autoantibodies, and histological data, the classification of inflammatory myopathy subtypes has been revisited progressively [9, 10, 11, 12, 13, 14]. Many cases previously considered as either polymyositis or dermatomyositis can now be assigned to more defined disease entities including inclusion body myositis, non-specific myositis, antisynthetase syndrome-associated myositis, and immune-mediated necrotizing myopathy. As a matter of fact, a diagnosis of polymyositis is becoming rare on the basis of histological criteria which are endomysial inflammatory T cell infiltrates surrounding and occasionally invading non-necrotic muscle fibers, or an endomysial CD8+ T cells surrounding but not definitely invading non-necrotic muscle fibers, or ubiquitous MHC class I expression [12, 15]. The designation “non-specific myositis” emerged from muscle biopsies that did not present the characteristics of either polymyositis or dermatomyositis [15], and most of the corresponding patients suffer from overlapping connective tissue disease. Pathological hallmarks of inclusion body myositis are intramyofiber vacuoles rimmed by basophilic protein inclusions and endomysial inflammation, indicating that the pathogenesis may have both a degenerative and an immune component [16]. Considering the clinical phenotype of dermatomyositis with the classical skin rash and depending on the present autoantibodies, the muscle biopsy may differ from the characteristic pattern which associates perifascicular atrophy highlighted by MHC class I immunopositivity, perivascular/perimysial inflammatory cell infiltrates [12], and depletion of intramuscular capillaries [17]. Ultrastructural evidence of tubuloreticular inclusions within endothelial cells of muscle capillaries occurs not only in dermatomyositis, but also in non-specific myositis especially in a setting of an associated connective tissue disease including systemic sclerosis, systemic lupus erythematosus, and Sjögren syndrome [18]. The aim of the present review is to clarify the contribution of muscle biopsy in a setting of idiopathic inflammatory myopathies overlapping with systemic diseases. Correlations between autoantibodies, histological subtypes on muscle biopsy, and clinical phenotypes are presented in Table 1. 

## Syndromes associated with myositis-associated autoantibodies 

**Figure 1.**
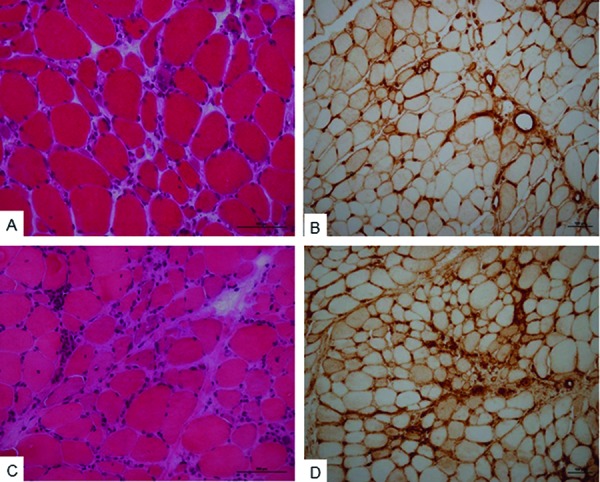
A, B: Muscle biopsy of a patient harboring both anti-PM/Scl and anti-Ku autoantibodies. This 60-year-old Caucasian man suffered from an overlap syndrome with myositis, systemic sclerosis, interstitial lung disease, and Raynaud phenomenon. Hematoxylin-stained frozen section (A) shows variations in myofiber sizes with a few regenerating fibers and mild endomysial lymphocytic infiltration. MHC class I immunostaining (B) is moderate on scattered myofibers. C, D: Muscle biopsy of a 25-year-old African woman harboring anti-U1snRNP and anti-Ro autoantibodies. She suffered from “mixed connective tissue disease” with myositis, systemic lupus erythematosus, rheumatoid arthritis, interstitial lung disease, pericarditis, Raynaud phenomenon, and puffy fingers. Hematoxylin-stained frozen section (C) shows variations in myofiber sizes and moderate endomysial lymphocytic infiltrates. MHC class I immunostaining (D) highlights several scattered myofibers.

Some of these MAAs, namely anti-SSA (Ro 60, Ro 52), anti-SSB (La), anti 75- and 100-kDa subunits of the nucleolar exosome complex (PM/Scl), and antibodies against 70- and 80-kDa subunits of a DNA-binding protein involved in double-stranded DNA repair (Ku) are commonly detected in patients suffering from idiopathic inflammatory myopathies associated with connective tissue diseases. Anti-PM/Scl and anti-Ku are also associated with an increased risk of ILD [3, 5]. Systemic sclerosis is the most common connective tissue disease associated with inflammatory myopathies, but there is still no agreement on this manifestation to be a true overlap or rather a complication related to systemic sclerosis itself [19, 20, 21]. Reports on muscle biopsy are heterogeneous considering inflammatory cell infiltrates [19, 21, 22, 23], whereas the prominence of fibrosis [21, 24] and necrosis [19, 22, 23] had been reported. The presence of fibrosis has been reported to be strongly associated with PM/Scl autoantibodies [23]. In a series of 11 patients harboring anti-Ku autoantibodies and presenting myositis, systemic sclerosis was associated in 5 cases, Sjögren syndrome in 2, and systemic lupus erythematosus in 1. Myofiber necrosis/regeneration and inflammatory cell infiltrates were reported in all cases except 1 with Sjögren syndrome, and a patient with systemic sclerosis presented inclusion body myositis-like features [25]. We present the muscle biopsy of a patient harboring both anti-PM/Scl and anti-Ku autoantibodies. This 60-year-old Caucasian man suffered from an overlap syndrome with myositis, systemic sclerosis, interstitial lung disease, and Raynaud phenomenon (Figure 1A, B). 

Although the occurrence of muscular symptoms in systemic lupus erythematosus is quite common, detection of inflammatory myopathy is rare. Reports of muscle biopsy data are few and limited to the description of perivascular mononuclear cell infiltrates and/or myofiber necrosis/regeneration [26, 27]. Rather numerous patients with Sjögren syndrome complain of pain or muscle weakness, but evidence of myositis is not frequent and only a few muscle biopsy reports are available. These describe perimysial and endomysial mononuclear cell infiltrates as well as myofiber degeneration/regeneration. An inclusion body myositis-like histopathological pattern has also been reported in association with Sjögren syndrome, the endomysial mononuclear cell infiltrates coexisting with rimmed vacuoles [2, 28, 29]. A recent paper reported 4 Sjögren syndrome patients who presented a common histologic pattern on muscular biopsy with germinal center-like structures resembling that present in salivary glands [30]. Myositis overlapping with rheumatoid arthritis appears unspecific with a variable degree of muscle fiber damage and inflammatory mononuclear cell infiltrates mainly perivascular and endomysial but also in the perimysial region [31, 32]. Occasional cases with an inclusion body myositis-like histopathological pattern have also been reported in association with rheumatoid arthritis [33, 34]. 

The term “mixed connective tissue disease” (MCTD) refers to a systemic autoimmune disease characterized by overlapping features between at least two systemic autoimmune diseases including systemic sclerosis, systemic lupus erythematosus, idiopathic inflammatory myopathy, and rheumatoid arthritis. Other clinical symptoms in MCTD include Raynaud phenomenon, puffy fingers, polyarthritis, interstitial lung disease, pleuritis, pericarditis, esophageal dysmotility, nervous system manifestations, and pulmonary arterial hypertension. If the presence of antibodies against the U1 small nuclear ribonucleoprotein autoantigen (U1snRNP) is considered as the serological hallmark of this condition [35, 36, 37, 38], the coexistence of other autoantibodies is common with significant influence on disease expression and clinical course. MCTD-associated myositis is generally subclinical, but severe muscle involvement and marked inflammation on biopsy may occur [39]. We illustrate the muscle biopsy of a 25-year-old African woman harboring anti-U1snRNP and anti-Ro autoantibodies. She suffered from “mixed connective tissue disease” with myositis, systemic lupus erythematosus, rheumatoid arthritis, interstitial lung disease, pericarditis, Raynaud phenomenon, and puffy fingers (Figure 1C, D). 

Anti-mitochondrial autoantibodies (AMA), directed against the 2-oxoacid dehydrogenase complexes existing in the inner membrane of mitochondria, are the characteristic markers of primary biliary cirrhosis. However, these autoantibodies may be found in association with other autoimmune disorders including systemic sclerosis, Sjögren syndrome, rheumatoid arthritis, and inflammatory myopathies. A statistical analysis revealed that inflammatory myopathies associated with AMA frequently include patients with a clinically chronic disease course, muscle atrophy, cardiopulmonary involvement, and granulomatous inflammation on muscle biopsy, regardless of the presence or absence of primary biliary cirrhosis [40]. 

Autoantibodies targeting the cytosolic 5’-nucleotidase 1A (cN1A) were frequently identified in patients with inclusion body myositis and have been expected to be a serological marker for early diagnosis [41]. However, anti-cN1A autoantibodies were also identified in other connective tissue diseases, especially systemic lupus erythematosus and Sjögren syndrome, but not in association with inclusion body myositis [42]. 

A myositis-overlap syndrome has been reported in 4 patients harboring autoantibodies to nuclear pore complexes (NPC). The clinical phenotype was characterized by prominent myositis in association with erosive arthritis, trigeminal neuralgia, mild ILD and Raynaud phenomenon [43]. In this report, the muscle biopsy data were considered consistent with inflammatory myopathy but without details. Further studies on large series of NPC seropositive patients with connective tissue diseases, and particularly patients with an overlap myositis syndrome are needed. 

## Syndromes associated with myositis-specific autoantibodies 

### Clinical overlaps of anti-synthetase syndrome associated myositis with specific autoantibodies 

**Figure 2.**
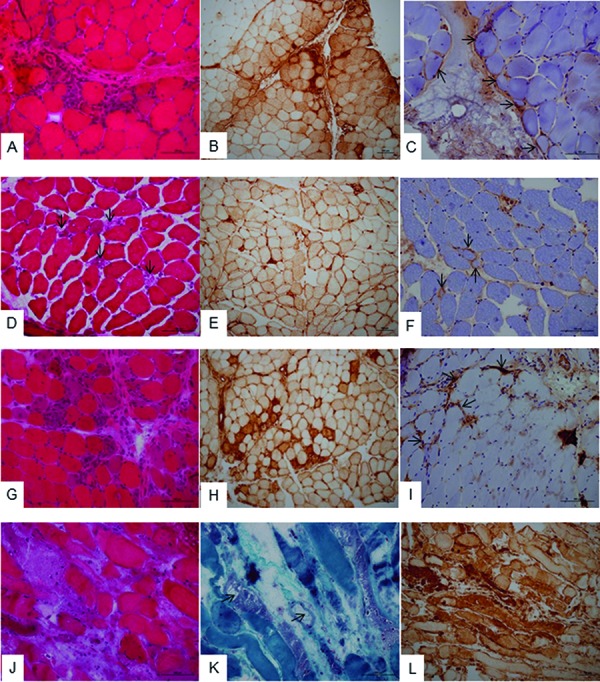
A, B, C: Muscle biopsy of a 47-year-old Caucasian woman harboring anti-Jo1 autoantibodies and suffering from an anti-synthetase syndrome with myositis, interstitial lung disease, and arthritis. Hematoxylin-stained frozen section (A) shows variations in myofiber sizes and necrotic fibers in the perifascicular area. MHC class I immunostaining (B) highlights the perifascicular lesions, and sarcolemmal C5-b9 complement deposition (C) is observed on the perifascicular area (arrows). D, E, F: Muscle biopsy of a 43-year-old Caucasian man harboring HMGCR autoantibodies and suffering from severe rhabdomyolysis but no extramuscular manifestation. Hematoxylin-stained frozen section (D) shows scattered necrotic myofibers with myophagocytosis (arrows), in the absence of significant lymphocytic infiltration. MHC class I immunostaining (E) is diffuse with focal enhancement around areas of myophagocytosis. The C5-b9 complement deposition (F) is patchy on the sarcolemma of some non-necrotic myofibers (arrows). G, H, I: Muscle biopsy of a 43-year-old Caucasian man harboring anti-Mi-2 autoantibodies and suffering from dermatomyositis with the characteristic skin rash. Hematoxylin-stained frozen section (G) shows perifascicular atrophy with necrosis and regeneration of myofibers, and lympho-macrophagic infiltrates. MHC class I immunostaining (H) highlights the perifascicular lesions, and the C5-b9 complement deposition (I) is observed on the capillaries (arrows). J, K, L: Muscle biopsy of a patient harboring anti-TIF1-γ and anti-Ro autoantibodies. This 80-year-old Caucasian man suffering from prostate and lung cancers presented an overlap syndrome associating dermatomyositis with edematous systemic sclerosis. Hematoxylin-eosin (J) and Gomori trichrome (K) frozen sections show an extensive necrosis of myofibers, non-rimmed vacuoles (arrows), and interstitial edema. Inflammatory cell infiltrates are not obvious. MHC class I immunopositivity of myofibers is extensive (L), and C5-b9 complement deposition on capillaries is masked by the strong and diffuse unspecific immunostaining of myofibers under necrosis (not shown). Bars = 100 µm.

The anti-synthetase syndrome (ASS) is defined by the presence of one of the anti-ARS autoantibodies in patients suffering from myositis, interstitial lung disease, arthritis, Raynaud phenomenon, fever, and a skin rash on the hands termed “mechanic’s hands” [4, 5, 6]. The combination of these hallmark signs may vary according to the type of the anti-ARS autoantibodies, anti-Jo1 being the more frequently encountered with a myositis dominant presentation. Other rarer anti-ARS associated with this syndrome include anti-PL7, anti-PL12, anti-EJ, anti-OJ, and anti-KS [44]. Anti-ARS autoantibodies are much less frequently found in juvenile myositis patients than in adults [3]. The characteristic histopathological pattern on muscle biopsy is the presence of perifascicular muscle fiber necrosis/regeneration and perimysial fragmentation of connective tissue [45, 46, 47, 48]. T- and B-lymphocytes, plasma cells and histiocytic macrophages are present in the perimysium and/or around vessels, with extension into the endomysium. MHC class I immunostaining is enhanced on the sarcolemma of perifascicular muscle fibers, and sarcolemmal C5b9 complement deposition is observed in the perifascicular areas [46, 47, 49]. By electron microscopy, myonuclear actin filament inclusions have been identified [47]. From several reports, ASS-associated myositis seems to be a separate entity rather than a variant of dermatomyositis or polymyositis [46, 49]. We show the muscle biopsy of a 47-year-old Caucasian woman harboring anti-Jo1 autoantibodies and suffering from an anti-synthetase syndrome with myositis, interstitial lung disease, and arthritis (Figure 2A, B, C). 

### Clinical overlaps of immune-mediated necrotizing myopathy with specific autoantibodies 

Most patients harboring anti-signal recognition particle (SRP) or anti-3-hydroxy-3-methylglutaryl-coenzyme A reductase (HMGCR) autoantibodies present the characteristic of immune-mediated necrotizing myopathy (IMNM). Not only adults but also juvenile patients can have anti-SRP or anti-HMGCR autoantibodies but at a lower frequency [3]. Muscular symptoms are usually severe with a sub-acute onset, but a slowly progressive course can occur and be difficult to differentiate from adult onset muscular dystrophies [14, 44]. Statin treatment has been regarded as a risk factor in patients with HMGCR autoantibodies [50]. Clinical features are shared between SRP-positive and HMGCR-positive patients, but muscle weakness is usually more severe in association with anti-SRP autoantibodies [51]. The frequency of extramuscular manifestations, including fever, skin rash, arthritis, Raynaud phenomenon, and mild ILD is low with both groups of autoantibodies, and a risk of malignancy has been demonstrated in both groups as well [51]. Muscle biopsy shows scattered necrotic and regenerative muscle fibers, whereas macrophages may be associated with necrotic fibers, but T cell infiltration is mild or absent. Sarcolemmal MHC class I immunostaining is diffuse with focal enhancement around areas of myophagocytosis. C5-b9 complement deposition may be observed patchy on the sarcolemma of non-necrotic muscle fibers and sparsely on endomysial capillaries [51, 52]. As already noticed, a combination of two MSAs remains occasional but it has been reported in a patient with anti-PL12 and anti-SRP, and suffering from ASS with IMNM [53]. We present the muscle biopsy of a 43-year-old Caucasian man harboring HMGCR autoantibodies and suffering from severe rhabdomyolysis but no extramuscular manifestation (Figure 2D, E, F). 

### Clinical overlaps of dermatomyositis with specific autoantibodies 

Patients harboring anti-nucleosome remodeling-histone deacetylase complex (Mi2), anti-melanoma differentiation-associated protein 5 (MDA5), anti-transcriptional intermediary factor 1 γ (TIF1-γ), anti-small ubiquitin-like modifier activating enzyme (SAE), or anti-nuclear matrix protein 2 (NXP2) autoantibodies present a clinical phenotype of dermatomyositis. 

Anti-Mi2 autoantibodies are more likely found in patients presenting characteristic dermatomyositis with heliotrope rash, shawl rash, Gottron papules and proximal weakness. Juvenile patients can have Mi2 autoantibodies but at a lower frequency than adults [3]. There is generally no other organ involvement and incidence of cancer is low although further investigation on more patients is necessary [44, 54]. The muscle biopsy harbors the characteristic lesions of dermatomyositis with the perifascicular atrophy and necrosis/regeneration of myofibers, lympho-macrophagic infiltrates within the perimysium and the endomysium. The lymphocytic populations are mainly T cells CD4+ and CD8+, but scattered B cells and plasma cells can also be present. MHC class I immunostaining highlights the perifascicular atrophy, and the C5-b9 complement deposition is observed on the sarcolemma and less frequently on capillaries [14]. We present the muscle biopsy of a 43-year-old Caucasian man harboring anti-Mi2 autoantibodies and suffering from characteristic dermatomyositis (Figure 2G, H, I). 

Patients with anti-MDA5 autoantibodies present a severe disease characterized by rapidly progressive ILD, skin ulcers, arthritis, fever, and a mild or even absent muscle involvement, called “amyopathic dermatomyositis” with either an adult or a juvenile presentation [55, 56]. Muscle biopsy is not characteristic as perifascicular atrophy is often lacking. Inflammatory infiltrates, consisting of macrophages and T cells, are absent or sparse and located mostly around scattered vessels in the perimysium. MHC class I expression is mild and limited to the sarcolemma of single myofibers. C5-b9 complement is not deposited on capillaries or the sarcolemma [14]. 

Anti-TIF1-γ autoantibodies are detected in juvenile and adult dermatomyositis with proximal limb weakness and commonly extensive skin manifestations. Extra-muscular involvement including ILD, Raynaud phenomenon, and arthritis remains uncommon, but a high incidence of association with malignancy has been reported in adult cases [56, 57, 58, 59]. Some patients present characteristic skin lesions including palmar hyperkeratotic papules, psoriasis-like lesions, hypopigmented and “red on white” telangiectatic patches [58]. In a series of 34 TIF1-γ-seropositive patients with cancer associated myositis, muscle biopsy showed frequent non-rimmed vacuolated myofibers, with less frequent necrotic/regenerating myofibers and perifascicular atrophy. Abundant mononuclear cell infiltrates were uncommon in the perimysium and not observed in the endomysium. The MHC class I immunopositivity was reported to be extensive on myofibers, and C5-b9 deposits dense on capillaries [59]. A recent paper described a similar histopathological pattern but underlined a severe edema [14]. We present the muscle biopsy of a patient harboring anti-TIF1-γ and anti-Ro antibodies. This 80-year-old Caucasian man suffering from prostate and lung cancers presented an overlap syndrome associating dermatomyositis with edematous systemic sclerosis (Figure 2J, K, L). 

The majority of patients with anti-SAE autoantibodies are adults presenting with the characteristic skin rash and clinically amyopathic dermatomyositis. Then they progress to develop myositis with a high incidence of severe dysphagia, and associated systemic features including fever and ILD [56, 60]. The association with cancer has been reported [61]. Muscle biopsy shows mild perifascicular atrophy and scarce lymphocytic infiltrates. MHC class I expression is scarce on the sarcolemma, and C5-b9 complement deposition is rare or absent on capillaries or myofibers [14]. 

Anti-NXP2 autoantibodies are rare, mostly found in juvenile dermatomyositis and strongly associated with severe muscle disease and skin extended calcifications [62, 63]. Adult cases present a high risk of cancer [57]. Muscle biopsy shows perifascicular atrophy and capillary dropout. There are CD4+ and CD8+ T cell infiltrates in the perimysium and occasionally in the endomysium. MHC class I immunostaining highlights the perifascicular atrophy, and C5b9 complement deposition is mild on myofiber sarcolemma as well as on capillaries [14]. 

## Conclusion 

Although the responsibility of autoantibodies themselves in the pathogenesis of idiopathic inflammatory myopathies is still uncertain, these may represent an increased risk of characteristic clinical associations. If MAAs are associated with non-specific myositis lesions in most cases, it is becoming evident that several MSAs are associated with more defined histopathological patterns on muscle biopsy. However, other autoantibodies remain to be discovered, and there appears to be clinical and histopathological variations depending on the coexisting autoantibodies. 

## Funding 

None. 

## Conflict of interest 

None. 

**Table 1.** Correlations between autoantibodies, histological subtypes on muscle biopsy, and clinical phenotype.

Myositis-associated antibodies	Histological subtypes on muscle biopsy	Clinical phenotype
Anti-Ro	Non-specific myositis	Various connective tissue diseases
Anti-La		
Anti-PM/Scl	Non-specific myositis	Various connective tissue diseases but most commonly systemic sclerosis, risk of interstitial lung disease
Anti-Ku	Non-specific myositis	Various connective tissue diseases, risk of interstitial lung disease
Anti-U1snRNP	Non-specific myositis	Mixed connective tissue disease: systemic sclerosis, systemic lupus erythematosus, rheumatoid arthritis, Raynaud phenomenon, puffy fingers, polyarthritis, interstitial lung disease, pleuritis, pericarditis, esophageal dysmotility, nervous system manifestations, pulmonary arterial hypertension
Anti-mitochondrial	Granulomatous myositis	Primary biliary cirrhosis, cardiopulmonary involvement, systemic sclerosis, Sjögren syndrome, rheumatoid arthritis
Anti-cN1A	Inclusion body myositis	Sjögren syndrome, systemic lupus erythematosus (not associated with inclusion body myositis)
Anti-NPC	Non-specific myositis	Erosive arthritis, trigeminal neuralgia, Raynaud phenomenon, mild interstitial lung disease
Myositis-specific autoantibodies	Histological subtypes on muscle biopsy	Clinical phenotype
Anti-aminoacyl-tRNA synthetases: anti-Jo1, anti-PL7, anti-PL12, anti-EJ, anti-OJ, anti-KS	Anti-synthetase syndrome associated myositis	Juvenile < adult Anti-synthetase syndrome: interstitial lung disease, arthritis, Raynaud phenomenon, fever, mechanic’s hands
Anti-SRP	Immune-mediated necrotizing myopathy	Juvenile < adult Extramuscular manifestations uncommon: fever, skin rash, arthritis, Raynaud phenomenon, mild interstitial lung disease Risk of cancer
Anti-HMGCR		
Anti-Mi2	Dermatomyositis	Juvenile < adult Characteristic skin rash Low risk of cancer
Anti-MDA5	Dermatomyositis	Juvenile and adult Mild or moderate/amyopathic dermatomyositis Rapidly progressive interstitial lung disease, skin ulcers, arthritis, fever
Anti-TIF1-γ	Dermatomyositis	Juvenile and adult Extensive skin manifestations Other extramuscular manifestations uncommon: interstitial lung disease, Raynaud phenomenon, arthritis High risk of cancer in adults
Anti-SAE	Dermatomyositis	Mainly adult Initially amyopathic Skin rash before the muscle disease Fever, interstitial lung disease Risk of cancer
Anti-NXP2	Dermatomyositis	Juvenile > adult Severe muscle disease Skin extended calcifications High risk of cancer in adults
